# Impact of COVID-19 on emotional and behavioral problems among preschool children: a meta-analysis

**DOI:** 10.1186/s12887-024-04931-8

**Published:** 2024-07-16

**Authors:** Jia-Qi Jing, Chang-Jiang Yang, Yue Wang, Xue-yun Su, Yi-Jie Du

**Affiliations:** 1 East China Normal University, 3663 N. Zhongshan Road, Shanghai, 200062 China; 2 Qingpu Traditional Chinese Medicine Hospital, 95 Qingan Road, Shanghai, 201799 China; 3 Academy of Integrative Medicine of Fudan University, 130 Dong’an Road, Shanghai, 200040, China

**Keywords:** COVID-19, Emotional disturbances, Behavioral problems, Preschool children, Meta-analysis

## Abstract

The impact of the Coronavirus Disease 2019 (COVID-19) on society is continuous, resulting in negative psychological consequences. Given the vulnerability and sensitivity to the environment among preschool children, their emotional and behavioral problems deserve more attention. The current study aimed to explore the impact of the epidemic on preschool children’s mental health by determining the pooled prevalence of emotional and behavioral problems amidst the Coronavirus Disease 2019 pandemic and to reveal potential reasons for variations between studies. Published studies were searched in Embase, PubMed, ProQuest, PsycINFO, Web of Science, CNKI, and Wanfang. Based on the inclusion criteria outlined in this study, a total of 10 studies encompassing 38,059 participants were incorporated. Employing a random-effect model for estimating the prevalence of emotional and behavioral problems, the results revealed a pooled prevalence rate of 24.3% (95% CI, 0.15–0.38; I²=99.9%) among preschool children. This rate surpasses the pre-outbreak prevalence observed in different countries, signifying a detrimental influence of the epidemic on the mental well-being of preschoolers. Therefore, mental health care and recovery are essential for the vulnerable group during and after the public health crisis. Specific emotional and behavioral problems among preschool children are expected to be researched in the future to provide more targeted guidance for intervention.

**Supplementary Information:**

The online version contains supplementary material available at 10.1186/s12887-024-04931-8.

## Introduction

The COVID-19 outbreak was declared a global public health emergency on January 30, 2020, by the World Health Organization [1], which continues to have an impact worldwide to date. In the wake of the enforcement of global epidemic prevention measures, the profound ramifications of isolation and uncertainty have emerged as formidable threats to individuals’ mental well-being, intensifying emotional disturbances marked by heightened levels of anxiety, depression, and stress [2]. In infants and preschool children, the susceptibility to environmental exposures renders them more vulnerable to severe physical consequences of COVID-19, resulting in systemic disease with several internal organ involvements [3, 4]. Prolonged lockdown-induced isolation may also lead to early-life mental health challenges in children, particularly manifesting as emotional and behavioral problems [3, 5]. Emotional and behavioral problems include a wide range of atypical behavior deviating from social standards [6], such as anxiety disorders, disruptive disorders, and oppositional defiant disorders [7], which are common psychiatric disorders during early childhood [8]. However, most existing research focused on children and adolescents, neglecting preschool children. Comparison of the prevalence of emotional and behavioral problems among preschool children before and during the pandemic could scientifically and intuitively reveal the impact of the epidemic on children’s emotional and behavioral health. Thus, it is essential to conduct a meta-analysis to estimate the prevalence of emotional and behavioral problems among preschool children during the outbreak, which is helpful to the intervention for emotional and behavioral disorders.

Before the pandemic, the prevalence of emotional and behavioral problems among preschool children ranged from 6.9 to 14.7% in various countries [9, 10]. During the pervasive lockdown, evidence suggested that the prevalence significantly elevated in countries worldwide, reaching up to 73.6% [11]. The following risk factors could explain preschool children’s increased emotional and behavioral problems. Firstly, a long-term lack of outdoor exercise may lead to the decreased release of certain neurotransmitters [12], such as low levels of dopamine, serotonin, and adrenaline [13]. The function of emotion regulation in the brain is then adversely affected [14]. Therefore, reduced movement behavior during the lockdown could trigger preschool children’s negative emotions through neural mechanisms [15, 16]. Secondly, caregivers’ perceived economic and parenting stress could induce insensitive parenting practices and negative family atmospheres [17]. Given the strong dependence of preschool children on the family environment, reduced parent-child closeness [18] and negative parenting attitudes may cause increased emotional and behavioral problems [8]. Thirdly, the developmental characteristics of preschool children could exacerbate the effect of the above factors. Children’s advanced cognitive functions are still developing; they may have difficulty monitoring their behavioral performance and regulating their emotions [19], which could affect preschoolers’ social adjustment [20]. Consequently, preschool children are more susceptible to the external environment [21], especially when faced with deviant stimuli. As such, exposure to adversity in early life could affect children’s brain structure and function [22], particularly in emotion regulation [23]. Therefore, it is critical to pay attention to preschool children’s emotional behavioral problems during the epidemic.

Moreover, emotional disturbances and behavioral problems in early childhood may lead to other severe consequences, specifically in social-emotional development and academic achievement. On the one hand, regarding social-emotional development, most children with emotional and behavioral problems were found to co-occur with language deficits [24, 25]. Developmental language disorders have an adverse impact on children’s communication skills [26, 27], thereby negatively affecting their social adjustment and life satisfaction. As the vital interactors of preschool children, parents’ emotions and interaction patterns are also affected by children’s emotional and behavioral problems [28]. Besides, evidence suggested that emotional and behavioral problems could result in inappropriate self-esteem and self-concept [29], which could predict antisocial personality, deliberate self-harm, and psychiatric problems in adulthood [8]. On the other hand, concerning academic achievement, numerous studies have shown that children with emotional and behavioral problems are at high risk of academic failure [30, 31]. The possible mechanism is children’s reduced attention to school work and school absence [32]. More seriously, it could lead to more unemployment, jobs held for a short time, lower job status and income, and other lifelong effects [8]. During the critical period of physical and psychological alternations, the negative consequences of emotional and behavioral problems are irreversible and have lifelong effects. Since recovery and inspiration plans for emotional and behavioral problems are under consideration [33], specific and precise estimates of children’s emotional and behavioral problems during the pandemic are essential, especially for preschoolers.

A growing number of empirical studies have been conducted to examine the prevalence of emotional and behavioral problems among preschool children during the COVID-19 pandemic. However, the prevalence of emotional and behavioral problems reported in studies varied considerably, ranging from 9.3 to 73.6% [11, 34]. Nevertheless, a study has shown that changes in problematic behaviors and anxiety symptoms among preschoolers were not statistically significant between the pre-pandemic and post-pandemic [26]. It is clear that current findings on the prevalence of emotional and behavioral problems among preschool children are inconsistent.

Hence, there is a necessity to conduct a meta-analysis of the prevalence of emotional and behavioral problems among preschool children in the context of the COVID-19 pandemic. Currently, only one meta-analysis is conducted to integrate the prevalence of emotional and behavioral problems. However, the participants were focused on children and adolescents instead of preschool children. Additionally, the above study was performed before the COVID-19 pandemic [35]. It follows from the above that no meta-analysis of the prevalence of emotional and behavioral problems among preschool children during the outbreak has been undertaken.

Therefore, the current study aimed to estimate the pooled prevalence among preschool children aged six years and under during the COVID-19 pandemic, reflecting the psychological effect of COVID-19. Moreover, we also examined potential factors that may explain the heterogeneity between studies. As such, the findings of our studies help to understand the prevalence of emotional and behavioral problems among preschool children during the COVID-19 pandemic and provide more information for further intervention and recovery.

## Method

### Study design and search strategy

The current study was conducted according to the Preferred Reporting Items for Systematic Reviews and Meta-Analyses (PRISMA) guidelines [36]. The study protocol was registered with the International Prospective Register of Systematic Reviews, PROSPERO (Protocol ID: CRD42022369811). As the current study exclusively utilized data from published studies and did not involve human or animal subjects, it fell outside the scope requiring ethical review.

Although emotional and behavioral problems contain multiple specific disorders, which are different clinically, most existing studies used comprehensive screening scales to evaluate children’s emotional and behavioral conditions due to the operational challenges of specific clinical screening during early childhood. The preliminary screening of emotional and behavioral problems could widely and conveniently reflect the psychological state of most preschoolers. Therefore, the current study chose emotional and behavioral problems as an indicator to reflect the epidemic’s impact on preschoolers. The search strategy in all databases included three themes: terms related to COVID-19, study population, and terms related to emotional and behavioral symptoms. The specific search terms were (‘COVID-19’ OR ‘SARS-CoV-2’) AND (‘child, preschool’ OR ‘pediatric’ OR ‘toddler’ OR ‘infant’) AND (‘emotional problem’ OR ‘behavioral problem’ OR ‘mental disorder’ OR ‘emotional and behavioral disorder’ OR ‘psychiatric’).

### Data sources

**Fig. 1:**
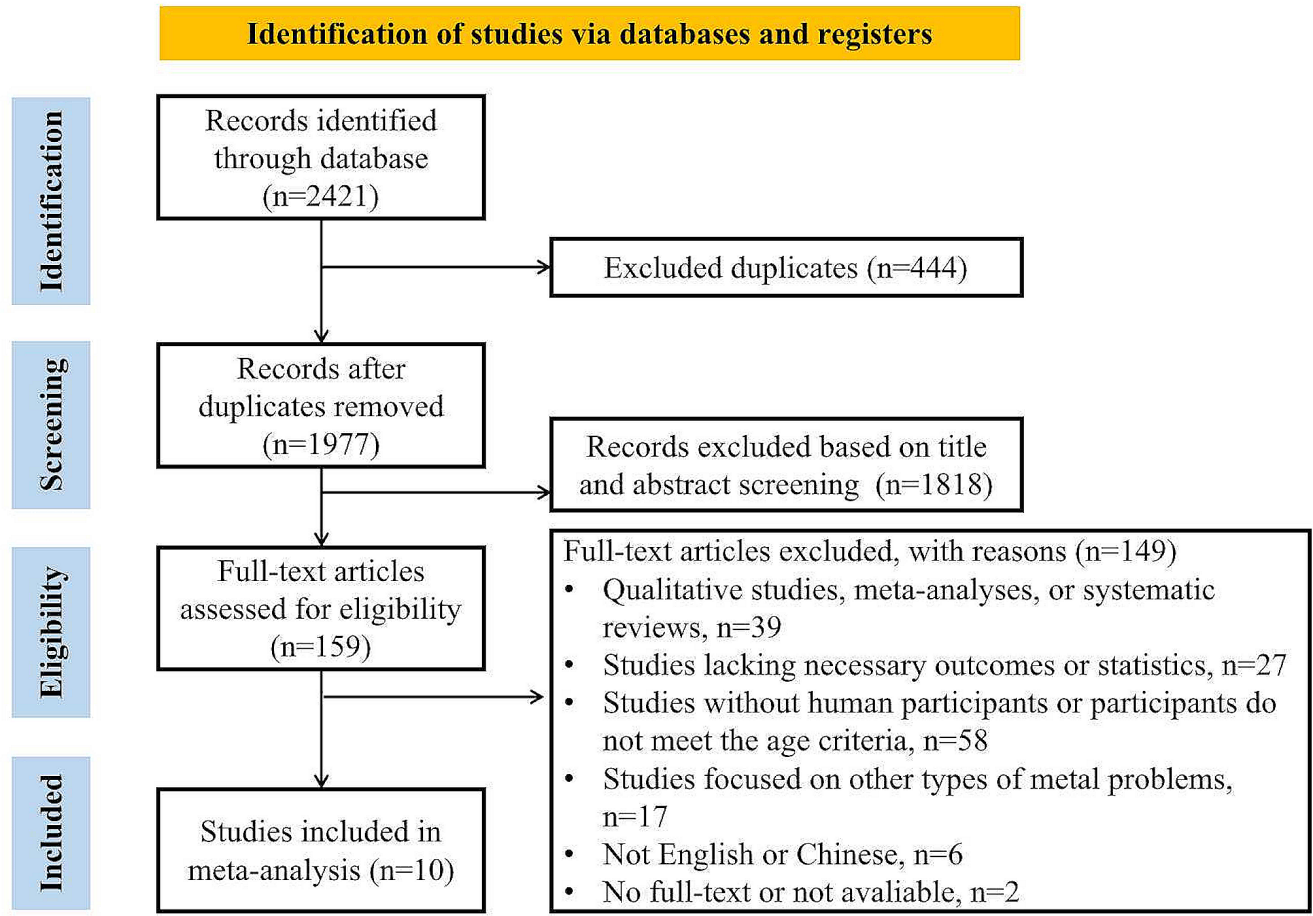
Flow chart of study selection

Literature searches were systematically performed in Embase, PubMed, ProQuest, PsycINFO, Web of Science, CNKI, and Wanfang databases, covering the period from their inception to June 1, 2023, to retrieve relevant literature pertaining to our research topic. Two authors independently searched the database with the search strategies and performed a supplementary manual search according to the references of the included studies to ensure good coverage. A total of 2421 studies were identified in the initial search process, including 444 duplicated studies. The complete flowchart of the study was presented in Fig. 1.

### Study selection

Considering the standard classification criteria across countries, the current study defines preschool children as children under 6 years old. Two authors autonomously screened studies based on the inclusion and exclusion criteria. The inclusion criteria included: (1) Cross-sectional or longitudinal study; (2) Studies measured preschool children’s emotional and behavioral problems with standardized assessment tools; (3) The participants were preschool children under 6 years or reported a separate prevalence at the age group; (4) Studies reported the rate or prevalence of emotional and behavioral problems; (5) Studies written in English or Chinese. The exclusion criteria included: (1) Qualitative studies, meta-analyses, reviews, editorials, case reports, or protocols; (2) Studies that did not provide the prevalence of emotional and behavioral problems or other necessary data; (3) Studies based on database analysis without human participants or participants did not meet the age criteria; (4) Studies focus on other types of mental problems; (5) Studies written in other languages.

### Data extraction

Two authors extracted data independently, and discrepancies were resolved via consensus. Prevalence of emotional and behavioral problems and potential moderators were extracted. For longitudinal studies, waves measured during or after the COVID-19 pandemic were chosen for extraction. Standardized data extraction was conducted from each article: author, publication year, geographical region, study design, sample size, the age of participants, number of children with abnormal scores, assessment tools, cutoff scores, and the reported prevalence.

### Quality and risk of Bias Assessment

The quality of each included study was assessed with the National Institute of Health Quality Assessment Tool for Observation Cohort and Cross-Sectional Studies [37] (Table 1 in Supplementary Material). The scoring criteria include sampling, objective outcome, exposure, and validity measures. If the answer is “yes”, the item is scored 1 point; if the answer is “no” “not clear” or “not applicable”, the item is scored 0 points. Studies were appraised as poor (total score < 4), fair (4 ≦ total score ≦ 6), or good (total score ≧ 7) methodological quality.

### Data analysis

The statistical analyses were conducted in R-5.4.1 statistical software, using “meta”, “metaphor”, and “robvis” packages. Pooled prevalence estimates were calculated using the random-effect model, considering the heterogeneity between studies. Given the premise of normal distribution, Logit transformation was used as a method of estimating the prevalence. Study heterogeneity was examined using the Q and I² statistics, with forest plots to visualize the results of meta-analyses. I² statistical estimates ≥ 50% indicated considerable statistical heterogeneity [38], suggesting potential sources of heterogeneity should be explored.

Subgroup analyses and meta-regression analyses were used to explore potential sources of significant heterogeneity. We selected geographical regions and assessment tools as categorical moderators, and sample size as a continuous moderator to explain the heterogeneity. Publication bias was examined by visual inspection of funnel plot symmetry, and the Egger’s test was conducted to determine the statistical significance. Besides, a sensitivity test was used to examine the presence of specific studies causing heterogeneity by excluding one article in each turn.

## Results

### Characteristics of included studies

A total of 2421 studies were found in the initial search results, of which 444 were duplicates. Based on the title and abstract review, 159 full-text articles were retrieved to screen according to the inclusion criteria, and 10 non-overlapping studies were eligible for meta-analyses **(**Fig. 1**)**. A total of 38,059 participants were included in studies, with a significant fluctuation of sample size between studies, from 61 to 16,094. The majority of studies were from East Asia (6 studies) [34, 39–43] with the rest of the studies from Europe (3 studies) [11, 44, 45] and North America (1 study) [46]. Nine studies were cross-sectional design, and one study was longitudinal design.

All studies investigated emotional and behavioral performance of preschool children through parent reports, using reliable and valid screening scales to measure emotional and behavioral problems in preschool children. The Strengths and Difficulties Questionnaire (SDQ) was used in five studies, the Preschool Pediatric Symptom Checklist (PPSC) and the Baby Pediatric Symptom Checklist (BPSC) were used in two studies, the Parent Symptom Questionnaire (PSQ) was used in two studies, and the Child Behavior Checklist (CBCL) was used once. The details of each included study were presented in Table 1.

**Table 1:** Characteristics of included studies

Author & publication	Country	Study design	Sample size	Age (range/mean ± SD)	Case	Assessment tools	Cutoff	Prevalence %	Study quality
1. Conti et al., 2020	Italy	Longitudinal	61	1.5-5y	31	CBCL1.5-5	T score ≧ 63	50.82%	Good
2. Oliva et al., 2021	Italy	Cross-sectional	7262	0–6 y	5344	BPSC&PPSC	> 9	73.59%	Fair
3. Wang et al., 2021a	China	Cross-sectional	8844	3–6 y/ 4.43 ± 0.98	1522	PSQ	Any factor score > the norm (2SD)	17.21%	Good
4. Wang et al., 2021b	China	Cross-sectional	504	3–6 y	79	SDQ	≧ 16	15.67%	Good
5. Wang et al.,2021c	China	Cross-sectional	16,094	0–6 y	2716	SDQ	≧ 16	16.87%	Good
6. Zhong et al., 2021	China	Cross-sectional	3029	3–7 y	282	SDQ	≧ 16	9.31%	Good
7. Zhu et al., 2021	China	Cross-sectional	1241	3–6 y	222	SDQ	≧ 16	17.89%	Good
8. Catherine et al., 2022	Germany	Cross-sectional	437	17–37 m/ 25.9 ± 6.52	37	SDQ	≧ 17	8.46%	Fair
9. Hails et al., 2022	America	Cross-sectional	267	1.5-5 y/ 3.43 ± 1.2	131	PPSC	> 9	49.06%	Good
10. Zhang and Chen, 2022	China	Cross-sectional	320	3–6 y/ 4.09 ± 1.10	69	PSQ	Any factor mean score > the norm	21.56%	Fair

### The pooled prevalence of emotional and behavioral problems

**Fig. 2:**
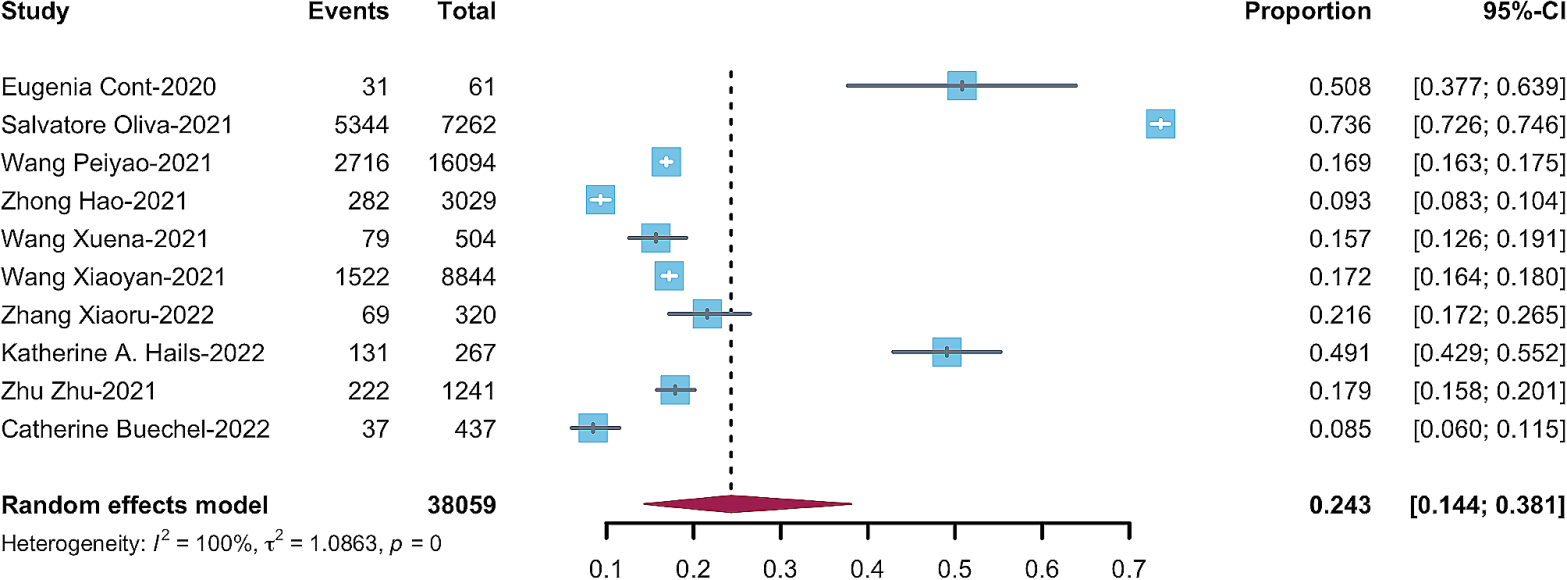
The pooled prevalence of emotional and behavioral problems among preschool children during the COVID-19 pandemic

Based on 10 included studies, the pooled prevalence from a random-effect meta-analysis of ten studies revealed a pooled prevalence of 24.3% (95%CI, 0.15–0.38; Fig. 2). The result showed significant heterogeneity between studies (I²=99.9%, Q = 7617.9, *p* < 0.010).

### Subgroup analysis and meta-regression analysis

**Fig. 3:**
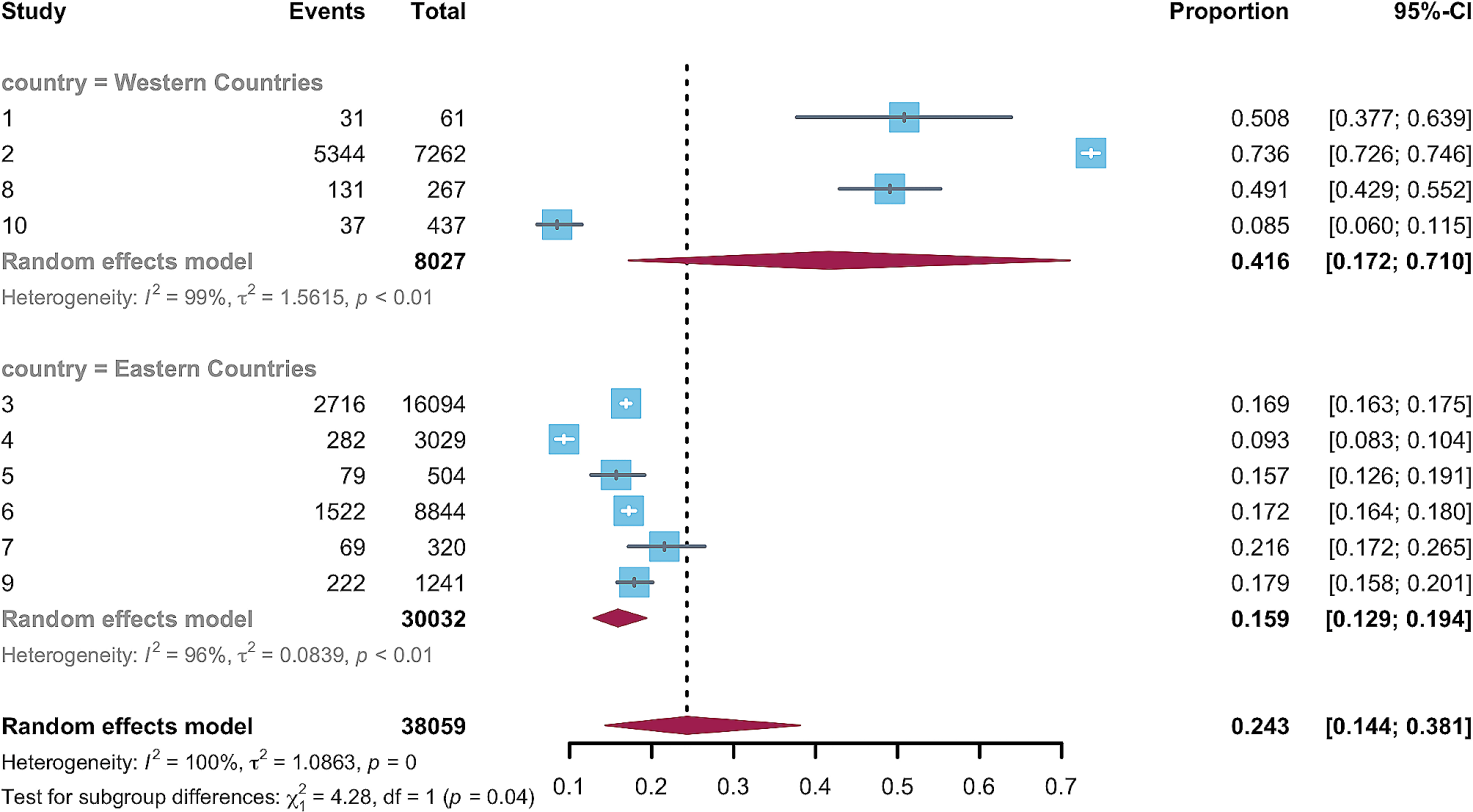
Subgroup analysis of the prevalence of emotional and behavioral problems among preschool children in different regions

**Fig. 4:**
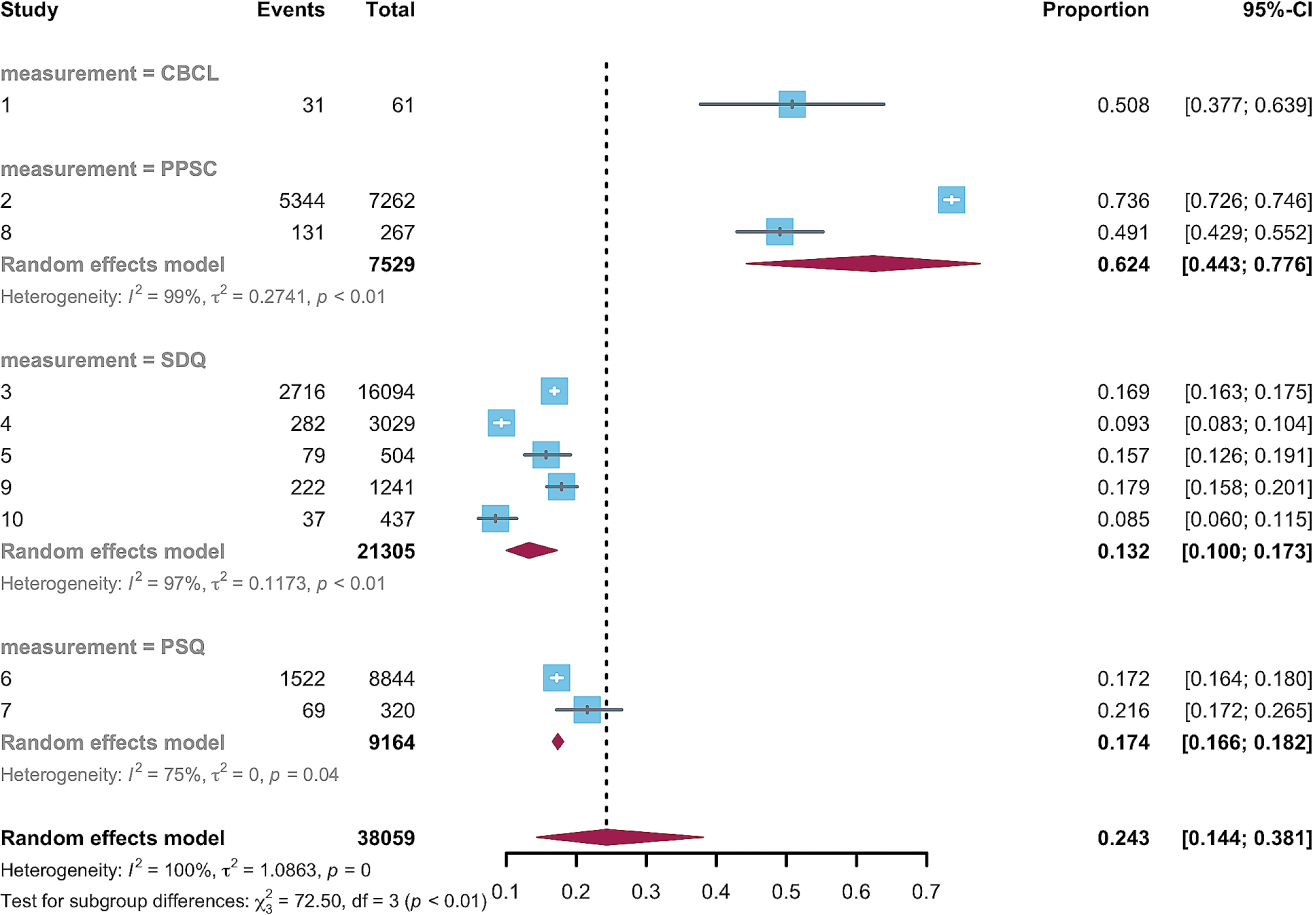
Subgroup analysis of the prevalence of emotional and behavioral problems among preschool children using different assessment tools

Given the significant heterogeneity, we conducted subgroup and meta-regression analyses based on the geographical region, assessment tools, and sample size. Two categorical moderators were both significant. The estimated prevalence of emotional and behavioral problems was 41.6% (95%CI, 0.17–0.71) in Western countries and 15.9% (95%CI, 0.13–0.19) in Eastern countries (Fig. 3). A meta-regression indicated significant differences between various study regions (*p* = 0.010). As for assessment, the estimated prevalence of studies using the PPSC (62.4%, 95%CI, 0.44–0.78) was significantly higher than that using the SDQ (13.2%, 95%CI, 0.10–0.17) and PSQ (17.4%, 95%CI, 0.17–0.18) (Fig. 4). A meta-regression indicated the moderating role of measuring tools is significant (*p* < 0.010). In terms of the continuous moderator, the meta-regression analysis indicated that the estimated prevalence of emotional and behavioral problems did not change with the sample size (*p* = 0.999).

### Quality assessment of the included studies

**Fig. 5:**
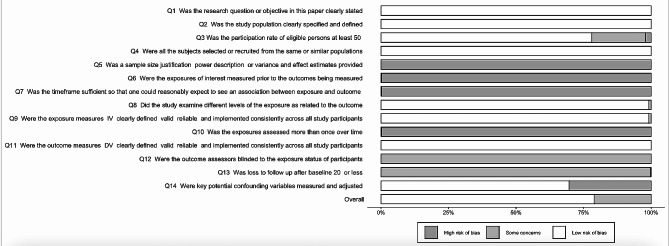
Number and percentage of included studies in each risk of bias of the National Institute of Health Quality Assessment Tool

As for the study quality, the overall quality of the included studies is satisfactory. Seven studies are good quality, and only three studies are fair. Apart from items that were not applicable due to the study design, the most common risk is the description of statistical power or effect estimates. A bias graph of the percentage of the included studies in each risk is presented in Fig. 5.

### Publication bias

**Fig. 6:**
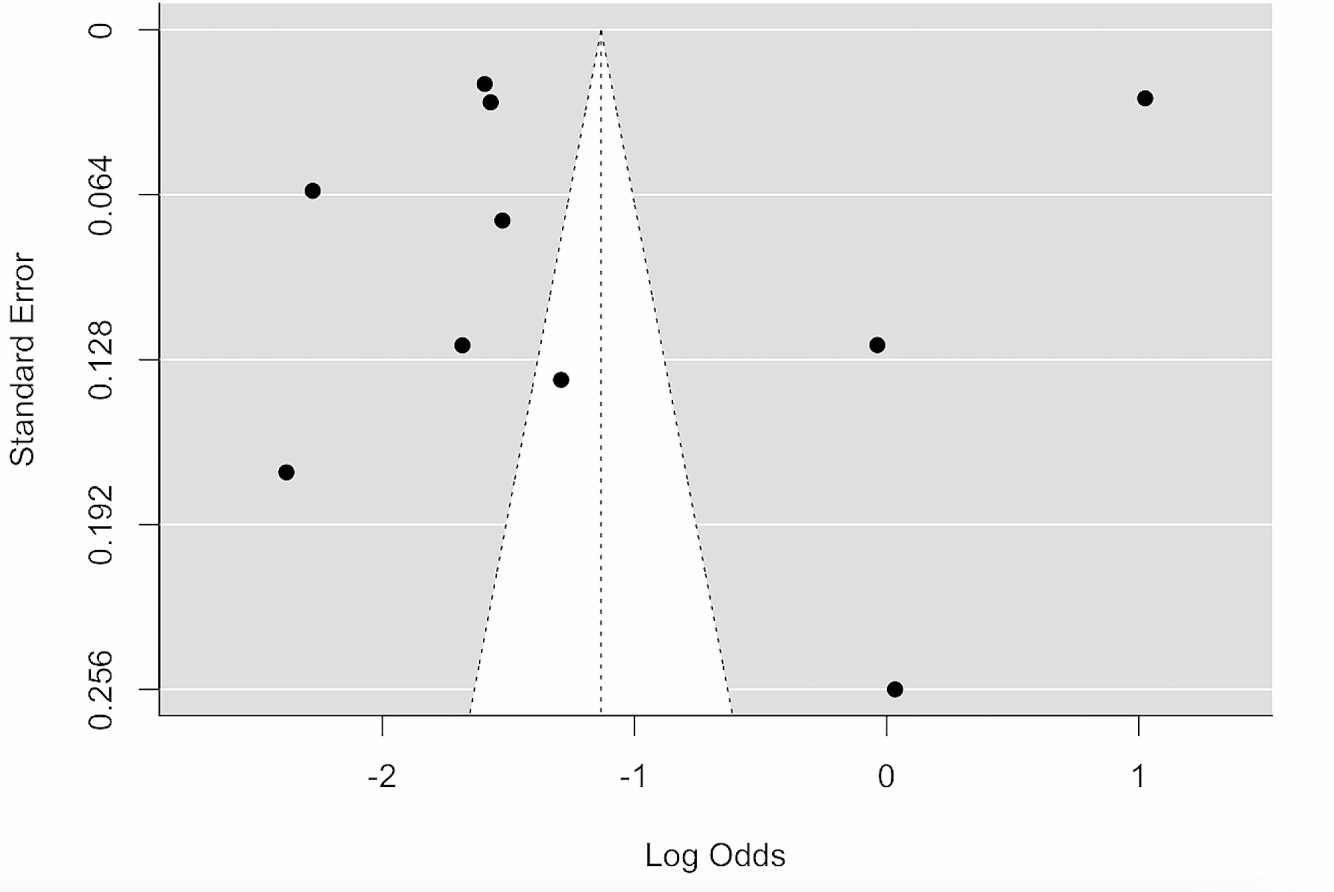
Funnel plot of prevalence of emotional and behavioral problems among preschool children during the COVID-19 pandemic

The visualization of publication bias was presented through the funnel plot (Fig. 6), which was slightly asymmetrical. More precisely, the Egger’s test indicated no significant publication bias (*p* = 0.811). As shown in the funnel plot, most of the scatters fall outside the triangular region, which is caused by the significant heterogeneity between studies.

### Sensitivity analysis

Sensitivity analysis by excluding each study individually suggested that the current meta-analysis was relatively reliable and stable. No study showed a variation of heterogeneity of more than 2% (Fig. 1 in Supplementary Material).

## Discussion

### Prevalence of emotional and behavioral problems among preschool children

The current meta-analysis was the first to explore the impact of COVID on emotional and behavioral problems among preschool children. Across 38,059 participants, the pooled prevalence of emotional and behavioral problems is 24.3%. The current findings indicated a threefold increase in prevalence compared to the pre-pandemic, such as 6.9% in Swiss [9], 7.1% in Norwegian [47], 7.4% in Germany, [48] and 9.8% in China [49]. According to publication bias and sensitivity analysis results, the study presented good reliability and stability. Therefore, there is a significant negative effect of COVID-19 on children’s emotional and behavioral health, in line with several longitudinal studies [50, 51].

The physiological mechanisms and psychological mechanisms of the pandemic on preschool children’s emotions and behaviors could explain the increased prevalence. Concerning physiological mechanisms, the cerebral cortex and neural network connections develop rapidly during the preschool period, while the brain is susceptible to environmental exposure. The physiological effects of the epidemic include disruption of biological rhythms and lack of physical activity. The disruption of biological rhythm refers to periodic disturbances in physiological and behavioral expression, embodying dis-regulated sleep among preschool children during the lockdown [52, 53]. Besides, children experienced increased screen time, increased sedentary behavior, and reduced outdoor activity due to epidemic prevention and control [54, 55]. The disruption of rhythm and lack of exercise influence the function of neurotrophic factors and neurotransmitters. Lack of exercise reduces protein expression of brain-derived neurotrophic factor (BDNF), insulin-like growth factor 1 (IGF-1), and vascular endothelial growth factor (VEGF), and deficiency of these neurotrophic factors negatively affects synaptic plasticity and neuronal survival, which in turn affects emotional processing and state stability through multiple signal pathways [56]. Similarly, the secretion of neurotransmitters such as serotonin, dopamine, and norepinephrine, which regulate anxiety and depression, is reduced due to lack of exercise [13, 57]. As important hormones for emotion adjustment, the variation could reduce feelings of euphoria and happiness and even lead to altered brain functions in the regulation of emotion and cognition [57]. Consequently, children who experienced detrimental living changes are likely to develop emotional and behavioral problems and other adverse psychological outcomes [16, 58, 59]. For instance, a study found that excessive screen time could impair the nervous system [60], which was linked to unfavorable temperaments [61], such as inattention and irritability, thereby resulting in children’s emotional disturbances and behavioral problems. Secondly, with respect to psychological mechanisms, the negative thoughts among preschool children and family stress triggered by the lockdown could be possible reasons. On the one hand, preschoolers’ developing cognitive capacities make the cognitive and behavioral responses toward stressful events more intense and sensitive. Existing findings indicated that preschool children worried about getting sick and permanent change and became scared of the pandemic [62, 63]. The worries and fear could lead to increased arousal, including irritability, sleep disturbance, and difficulties regulating emotions [63]. Studies have shown that the younger generation exhibited lower resilience and adaptability in dealing with stressful events, facing challenges in adjusting to the lifestyle changes imposed by the epidemic [64]. Consequently, their mental health status merits careful consideration [65, 66]. However, it is essential to acknowledge that the perception of stressful events varies among individuals and may be influenced by factors such as temperament, personality, family atmosphere, and past experiences. For instance, individuals who perceive their environment as predictable and maintain dynamic confidence in stressful events exhibit heightened adaptive capabilities and are more likely to buffer against mental symptoms induced by the epidemic [67]. On the other hand, as the “cumulative stress” hypothesis explains, the effects of stress exposure in early life are cumulative, making individuals more vulnerable to mental disease [68, 69]. As the direct external environment for early development, family pressure could adversely affect children’s emotions and behaviors. Parents with healthy emotional and psychological states can buffer children’s stresses and help them manage their feelings [70]. On the contrary, parents’ perceived life stress significantly increased because of the lockdown, leading to negative parenting. Frequently use of negative parenting strategies could predict preschool children’s behavioral disorders [26, 71]. Studies also found that maternal mood and harsh parenting mediate the increase in emotional and behavioral problems [72, 73]. Therefore, risk factors related to the epidemic influence preschoolers’ emotions and behaviors through physiological and psychological mechanisms.

### Prevalence of emotional and behavioral problems in different regions

In addition, the current study found that the potential sources of heterogeneity were study regions and measuring tools. The estimated prevalence in China was significantly lower than in Italy, Germany, and America. Consistent with the existing studies, a meta-analysis found that the prevalence of child posttraumatic stress disorder in China was significantly lower than the Italian and American estimates [74]. Firstly, the difference may be due to epidemic crisis degrees and prevention and control policies in different countries. Considering the data collection time, most of the included studies collected data after March 2020, with two studies in 2021. At that moment, the spread of the epidemic in China was contained generally, and people were gradually adapting to the prevention and control requirements [75, 76]. However, Western countries were faced with a critical period in fighting against the epidemic at that time, with broad and comprehensive social coverage. Accordingly, the perceived worry and stress could exacerbate emotional and behavioral problems. Thus, the phenomenon indirectly demonstrated the impact of the epidemic on the emotions and behaviors among preschool children. Secondly, society and cultural clusters could influence the discrepancy. A study indicated a social and cultural difference in the prevalence of parent-report emotional and behavioral problems among children and adolescents [77]. Specifically, under some cultural beliefs, people are reluctant to endorse mental diseases because of the fear of being regarded as abnormal. Besides, parents may be less sensitive to children’s emotional and behavioral changes in children during the outbreak, resulting in underreporting in China. Notably, the degree of the epidemic crisis elevated significantly in 2022 in China, followed by strict prevention and control policies. In contrast, most Western countries present satisfactory control of the epidemic currently. Further research should focus on the impact of more recent outbreaks on preschoolers’ development, and the findings may differ from the current findings.

### Prevalence of emotional and behavioral problems using different assessment tools

Four screening scales were used in the included studies, which could be the cause of the heterogeneity. The results indicated a higher rate of emotional and behavioral problems in studies using PPSC than in the SDQ and PSQ. The composition and characteristics of different instruments may explain the result. Firstly, different ranges of abnormal behaviors covered by assessment tools could lead to heterogeneity. Although the CBCL has solid psychometric properties [78], the length of the instrument (100–119 items per form) places a burden on researchers. With 25 items in total, the SDQ is simple and convenient to assess and score, showing good reliability and validity [79, 80]. However, the CBCL is far more comprehensive than the SDQ. For instance, only one item on somatic complaints was included in the internalizing domain of the SDQ, while the CBCL includes a whole subscale of somatic complaints [81]. Therefore, the SDQ might omit common behavioral problems, resulting in a lower reporting rate. Similarly, the PSC shows reasonable specificity and sensitivity [82], while it is mainly used for screening attention deficit hyperactivity disorders. Thus, the scale is more reliable when measuring specific syndromes or difficulties. Several symptoms may be missed when measuring a wide range of emotional and behavioral disorders, resulting in a lower prevalence in the current study. Secondly, parent-report could be vulnerable to confounding factors. The new domain in the PPSC and BPSC, parenting challenges [83], may account for the high prevalence of emotional and behavioral problems. Although studies have shown that children’s emotional and behavioral problems could increase parenting stress [84], parenting stress could also be influenced by complex factors during the pandemic, such as unemployment and economic hardship. Accordingly, the reported prevalence of emotional and behavioral problems might be higher due to significant perceived parenting challenges. Thus, whether there is an actual difference in efficiency between these scales should be explored further.

### Limitation

There are several limitations in the current study. Firstly, one critical limitation is that the number of included studies was relatively small and the majority of studies were conducted in China. Consequently, the combined prevalence and heterogeneity could be affected. Secondly, the lack of longitudinal studies makes it difficult to accurately determine whether the lockdown has a sustained impact on preschoolers’ emotional and behavioral problems. Thirdly, we found a substantial heterogeneity between studies. Although the heterogeneity could be explained by study regions and assessment tools, the above moderators should be further explored. Finally, screening for emotional and behavioral problems is broad, covering different abnormal psychological traits. Research on the epidemic’s impact on children’s psychological problems remains cursory in screening. There is a gap in exploring specific emotional and behavioral problems among preschoolers, such as anxiety, depression, aggression, and oppositional defiant disorder. Further empirical research and meta-analyses should be conducted on the effects of epidemic or environmental exposures on specific disorders, which could provide a more targeted reference to clinical intervention and recovery.

## Conclusion

Overall, the meta-analysis suggested increased emotional and behavioral problems among preschool children during the COVID-19 pandemic, with an estimated prevalence of 24.3%. The combined prevalence of Western countries was significantly higher than Eastern countries. The findings indicated that preschool children’s emotional and behavioral problems should be attached to great importance after the epidemic. Accordingly, intervention and recovery should be carried out to improve preschool children’s mental health globally.

### Electronic supplementary material

Below is the link to the electronic supplementary material.

Supplementary Material 1

Supplementary Material 2

## Data Availability

The data that support the findings of the article are openly available at 10.7910/DVN/KIFDWR.
