# Supplementary figures and images for: Impact of COVID-19 on emotional and behavioral problems among preschool children: a meta-analysis
Source: BMC Pediatr. 2024 Jul 16;24:455. doi: 10.1186/s12887-024-04931-8 (PMC11251369; doi:10.1186/s12887-024-04931-8)

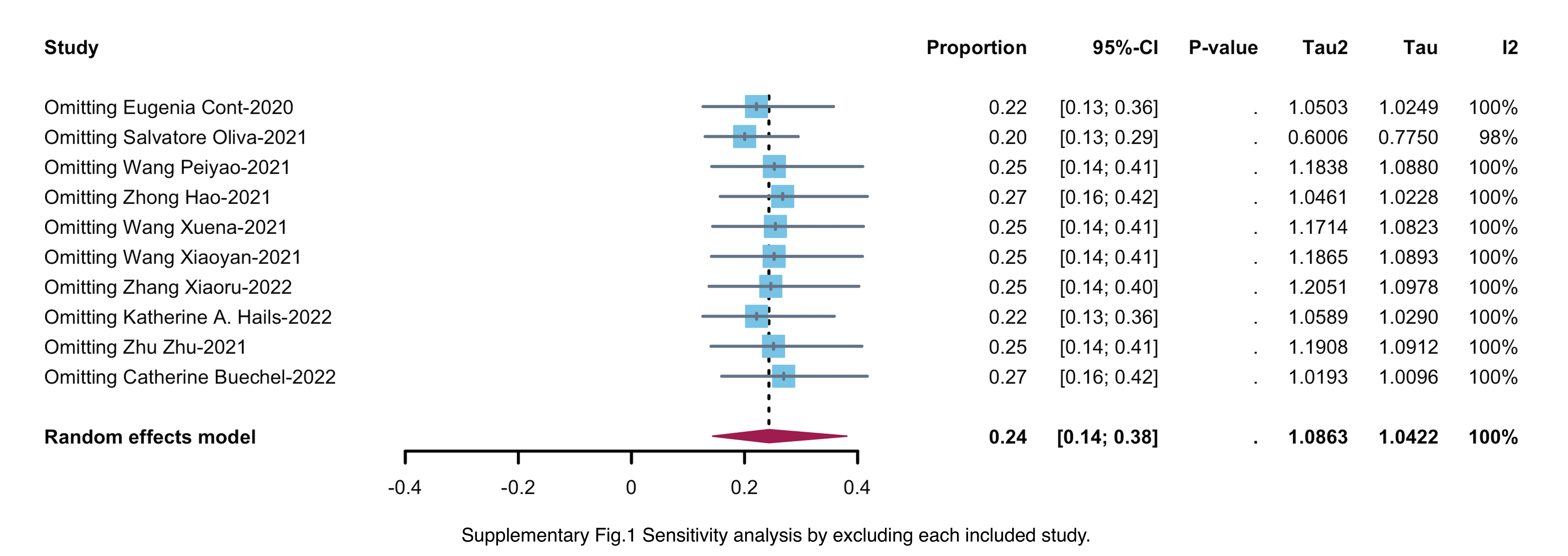

Supplement: Supplementary file 2 — Supplementary Material 2 [file 12887_2024_4931_MOESM2_ESM.png]
